# Reverse Sural Flap Venous Congestion Successfully Managed With Enoxaparin in a Male Patient With a Traumatic Foot Crush Injury: A Case Report

**DOI:** 10.7759/cureus.101926

**Published:** 2026-01-20

**Authors:** Jonathan Velasco-Bustamante, Dagmar Velasco-Bustamante, Cecibel Bravo-Romero, Cesar Nacimba-Aman, Wilson S Peñafiel-Pallares

**Affiliations:** 1 Plastic and Reconstructive Surgery, Pontificia Universidad Católica Del Ecuador, Quito, ECU; 2 Plastic and Reconstructive Surgery, Pontificia Universidad Católica del Ecuador, Quito, ECU; 3 General Practice, Universidad de las Américas, Quito, ECU

**Keywords:** enoxaparin, lower-extremity reconstruction, low-molecular-weight heparin, reverse sural flap, salvage therapy, venous congestion

## Abstract

The reverse sural flap is a commonly used surgical option for soft-tissue defects of the distal leg, ankle, and heel in hospitals where microsurgical capabilities are limited. However, one of its most frequent complications is venous congestion, which can lead to partial or total flap necrosis if not promptly addressed. We present the case of a patient with a traumatic crush injury of the right ankle, reconstructed with a reverse-flow sural flap that subsequently developed venous congestion in the immediate postoperative period (<12 hours). A local subcutaneous enoxaparin protocol (low-molecular-weight heparin) was implemented as a salvage strategy. This intervention successfully reversed the clinical signs of venous congestion and preserved approximately 80% of the flap's viability. This report discusses the underlying pathophysiology, the applied protocol, previous evidence, and the clinical implications of this approach.

## Introduction

The reconstruction of soft-tissue defects in the middle and distal thirds of the leg and the ankle region remains a persistent challenge for plastic surgeons. Although microvascular free flaps often provide excellent outcomes within the reconstructive ladder, they require specialized infrastructure, longer operative times, and plastic surgeons with dedicated microsurgical training. In settings with technical, anatomical, or economic limitations, well-designed pedicled flaps continue to serve as reliable workhorse options for these types of defects [1-3].

The reverse sural flap is one such pedicled flap of choice, particularly for defects located in the middle and distal thirds of the leg, the malleolar region, the heel, and the distal foot. Its design relies on retrograde vascularization supported by perforating branches of the peroneal artery and the superficial venous system, including the lesser saphenous vein and the sural venae comitantes [4,5]. One of its primary advantages is that it does not require microvascular anastomosis, thereby decreasing operative time and eliminating the need for microsurgical equipment [6]. Although multiple salvage strategies have been described, there is no universally accepted standardized approach for managing early venous congestion in pedicled flaps.

However, the success of this flap depends largely on adequate venous drainage. Venous congestion is a very common and challenging complication because impaired venous return leads to progressive stasis, edema, microvascular thrombosis, and ultimately flap necrosis if not promptly addressed. Clinically, venous congestion typically manifests within the first 6 to 12 postoperative hours and is characterized by dark violaceous discoloration, increased swelling, delayed capillary refill (>2 seconds), marked vascular turgor, temperature asymmetry (>2°C difference), and dark bleeding upon puncture.

When venous congestion occurs, intervention must be rapid and effective. Widely used strategies include surgical re-exploration (pedicle release, correction of torsion or suture-related compression), mechanical decompression, venocutaneous drainage, hirudotherapy (medicinal leeches), and pharmacologic therapies with anticoagulants [4].

Among anticoagulants, enoxaparin, a low-molecular-weight heparin (LMWH), has been proposed as an effective alternative when surgical or biological methods are unavailable. Pérez et al. described a protocol involving local subcutaneous enoxaparin administration in congested flap areas, reporting encouraging results [7].

This case is noteworthy because it illustrates the successful salvage of a reverse sural flap with early-onset venous congestion using a structured local subcutaneous enoxaparin protocol, without the need for microsurgical intervention or medicinal leeches. The protocol was implemented promptly within the first postoperative hours and adapted according to flap surface area and clinical evolution, allowing preservation of approximately 80% of flap viability. This case report was prepared and reported in accordance with the SCARE 2020 guidelines for surgical case reports.

## Case presentation

**Figure 1:**
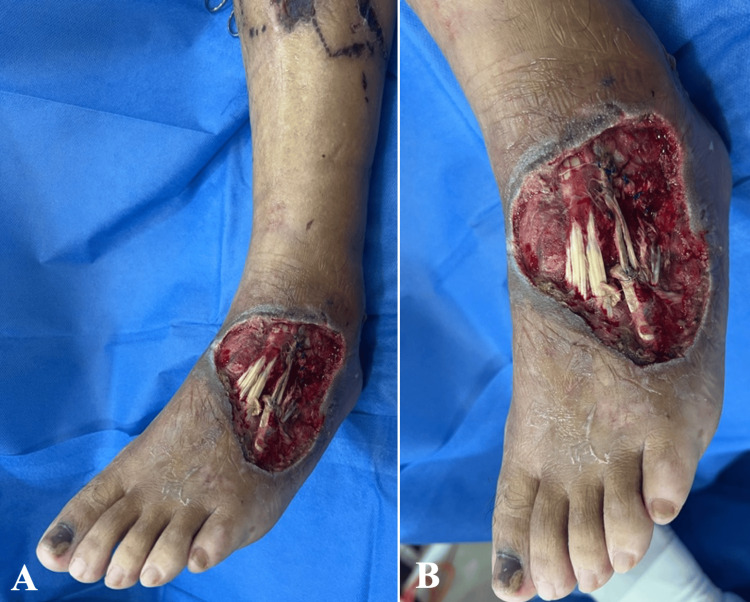
Soft-tissue defect of the left dorsal foot with exposed tendons. (A, B) Soft-tissue defect located on the proximal-lateral dorsal aspect of the left foot, measuring approximately 10 cm in diameter, with exposure of the extensor tendons.

A 58-year-old male patient with no significant past medical or surgical history and no history of smoking, alcohol, or recreational drug use, presented to the emergency department of a tertiary care hospital after being brought by ambulance following a crush injury involving the proximal and lateral aspects of the dorsum of the left foot. The injury occurred during a work-related accident in which the patient’s left foot was crushed by heavy machinery. Physical examination revealed an area of soft-tissue necrosis measuring approximately 10 cm in diameter, with exposure of the extensor digitorum longus tendon (Figure 1).

During hospitalization, the patient underwent advanced wound care, as well as operative debridement of devitalized tissue using both surgical and mechanical techniques. Wound cultures were obtained before definitive coverage. After five days of inpatient management and following negative culture results (no bacterial growth), the case was reviewed, and soft-tissue reconstruction using a reverse sural flap was planned in accordance with the reconstructive ladder. A complete timeline of the clinical course and key events is presented in Table 1.

**Table 1:** Timeline of the patient’s clinical course, surgical management, and local subcutaneous enoxaparin salvage protocol.

Time	Event
Day 0	Traumatic crush injury
Day 5	Reverse sural flap performed
+12 h	Venous congestion detected
Day 1-3	Local subcutaneous enoxaparin 20 mg every 4 hours
Day 4-6	Local subcutaneous enoxaparin 10 mg every 8 hours
Day 7-9	Local subcutaneous enoxaparin 10 mg every 12 hours
Day 10-14	Local subcutaneous enoxaparin 10 mg every 24 hours; tangential debridement performed
Day 28	Split-thickness skin graft
Day 53	Complete epithelialization

The patient was positioned in the prone position. Under spinal anesthesia, a midline was drawn from the lateral malleolus toward the mid-portion of the popliteal fossa, and the leg was divided into three thirds. A skin island measuring approximately 10 cm in diameter was designed at the junction of the upper and middle thirds, with the medial, lateral, and inferior borders of the gastrocnemius muscle serving as anatomical limits. The pivot point was marked approximately 5 cm proximal to the lateral malleolus.

Dissection began at the distal border of the flap. After incising the deep fascia and exposing the gastrocnemius muscle bellies, the medial sural nerve and the lesser saphenous vein were identified; the vein was ligated proximally. Dissection was then continued in a subfascial plane from proximal to distal, maintaining a pedicle width of 4 cm up to the pivot point, which was located 5 cm above the lateral malleolus.

**Figure 2:**
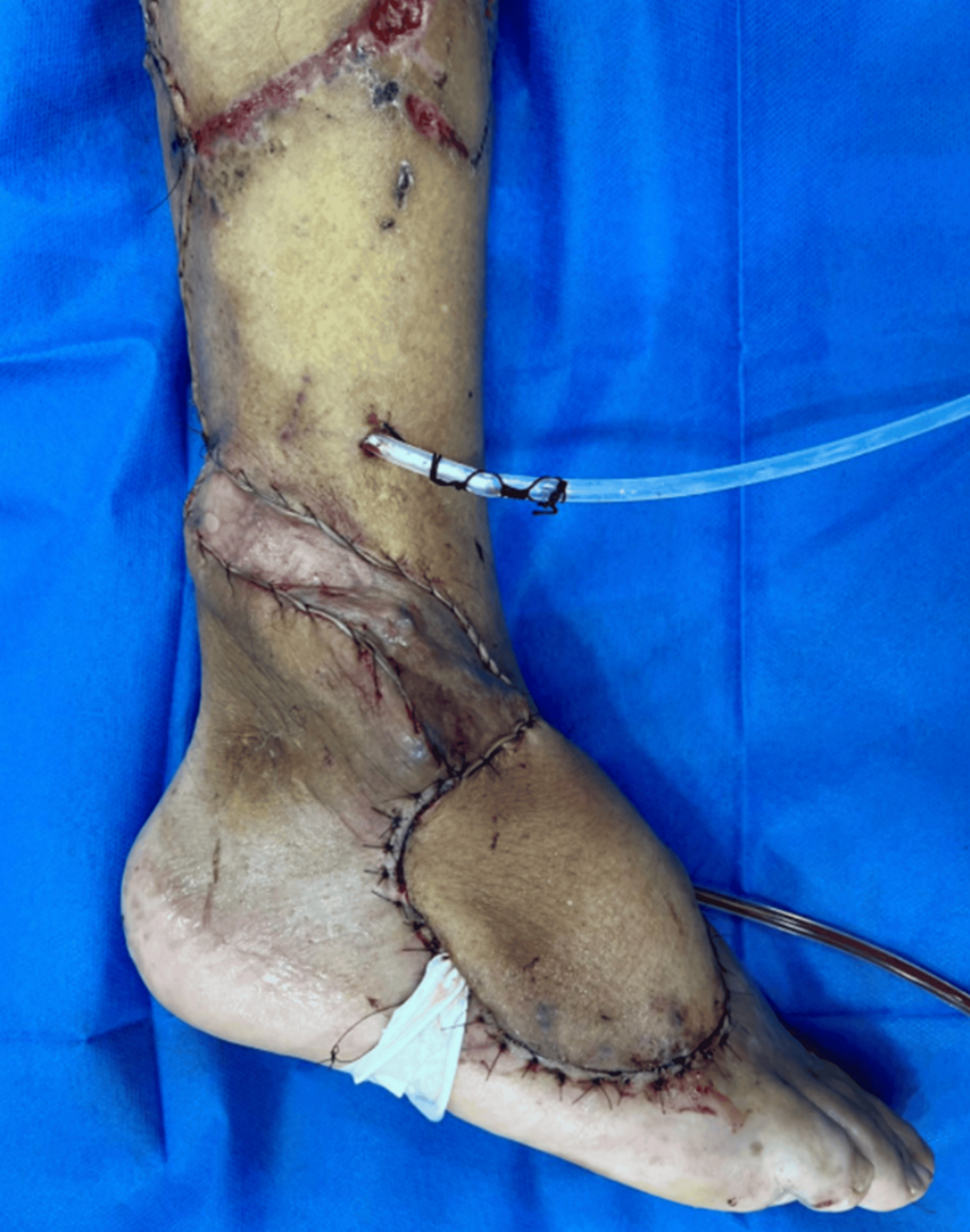
Postoperative placement of the reverse sural flap. Immediate results of the reverse sural fasciocutaneous flap rotated and inset to cover the dorsal foot defect.

The reverse sural flap was subsequently rotated 180° to cover the defect. Fixation was performed with 3-0 nylon sutures, and a capillary drain was placed in the recipient site, along with a suction drain in the donor area. The immediate postoperative appearance of the flap is shown in Figure 2.

At 12 hours postoperatively, the patient developed distal venous congestion characterized by dark violaceous discoloration, capillary refill time greater than 3 seconds, increased vascular turgor, cool temperature, and dark blood upon needle puncture. At that time, the patient was hemodynamically stable, with normal vital signs. In response, several inset sutures were removed, the leg was elevated and immobilized to optimize venous drainage and minimize edema, and a 5-cc hematoma was evacuated.

Given the absence of alternative salvage options, such as hirudotherapy or microsurgical venous revision, in our institutional setting, a salvage protocol using local subcutaneous enoxaparin was initiated according to the technique described by Pérez et al. for managing flap venous congestion with LMWH as a chemical leech [7].

Pérez et al. described a reproducible clinical protocol for the local subcutaneous administration of enoxaparin in flaps complicated by venous congestion, establishing dosing, frequency, incision technique, and duration of treatment [7]. The recommended regimen varies according to the extent of the affected area; therefore, in the present case, it was applied as follows: during postoperative days 1 to 3, the patient received 20 mg of local subcutaneous enoxaparin every 4 hours. Between postoperative days 4 and 6, the dose was reduced to 10 mg every 8 hours, followed by 10 mg every 12 hours on days 7 to 9, and finally 10 mg every 24 hours from days 10 to 14 [7].

Superficial skin incisions were performed before each application to facilitate the drainage of congested blood and improve local venous outflow. In addition, hemoglobin levels were monitored every 24 to 48 hours, and transfusions were administered when clinically indicated. The protocol was adjusted according to the patient’s clinical evolution.

**Figure 3:**
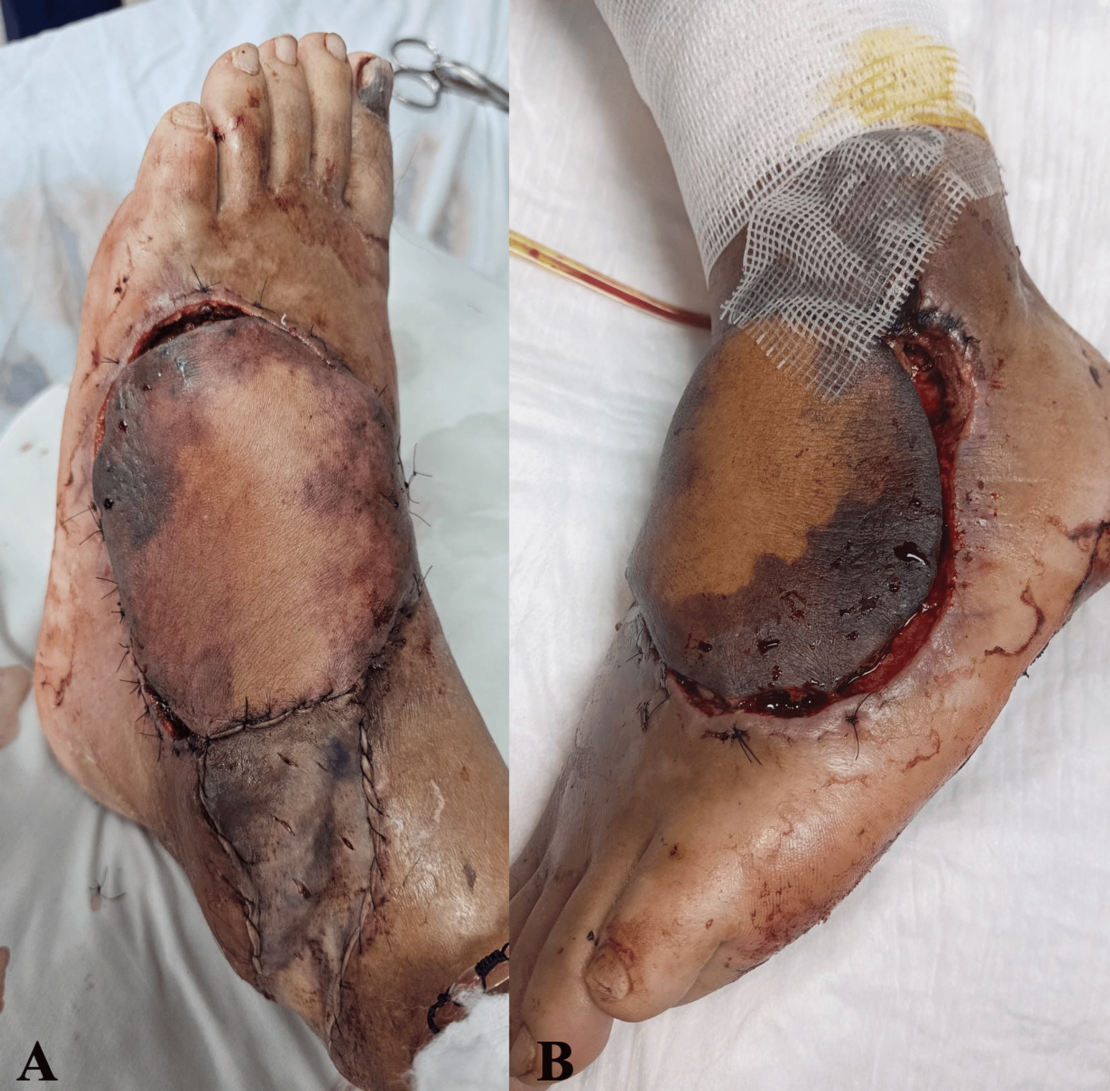
Evolution of venous congestion during the enoxaparin salvage protocol (A) Postoperative day 1: initiation of local subcutaneous enoxaparin every 4 hours due to clinical signs of venous congestion. (B) Postoperative day 4: enoxaparin adjusted to every 8 hours, with partial improvement of venous congestion.

During the first three postoperative days, the congested area measured less than 75 cm²; therefore, enoxaparin was administered at a dose of 20 mg every 4 hours (Figure 3A). This dosing strategy facilitated adequate venous drainage and reduced blood stasis, thereby improving the flap’s microcirculation.

**Figure 4:**
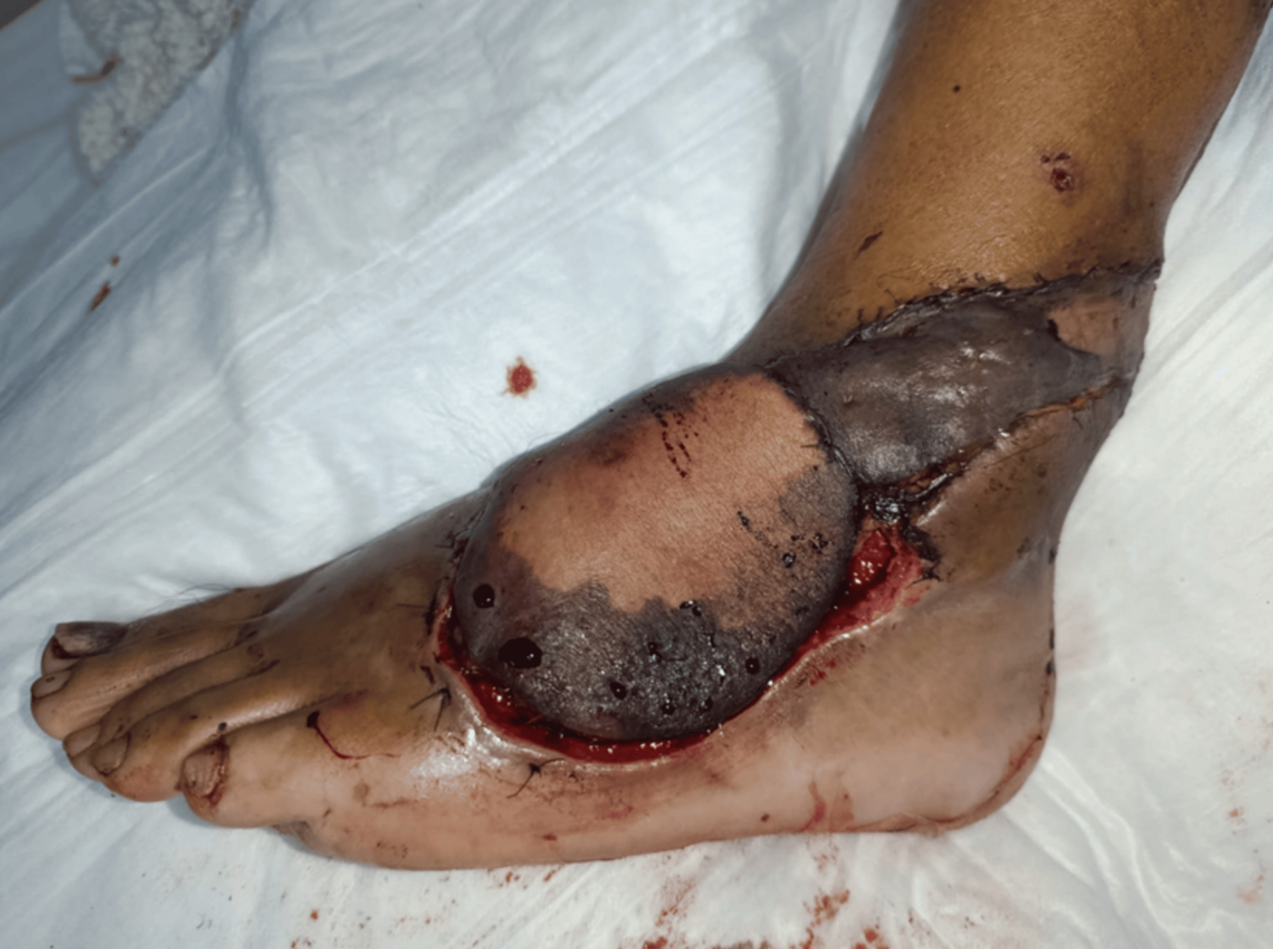
Postoperative day 7 during the enoxaparin salvage protocol. Enoxaparin was administered every 12 hours. Early demarcation of necrotic areas was observed.

Between postoperative days 4 and 6, the enoxaparin dose was reduced to 10 mg every 8 hours, as established in the protocol. This adjustment resulted in continued improvement in flap circulation, with clinical signs of enhanced perfusion and decreased congestion (Figure 3B). From postoperative days 7 to 9, the dose was further reduced to 10 mg every 12 hours, reflecting clinical stabilization of the flap and a positive response to treatment (Figure 4).

**Figure 5:**
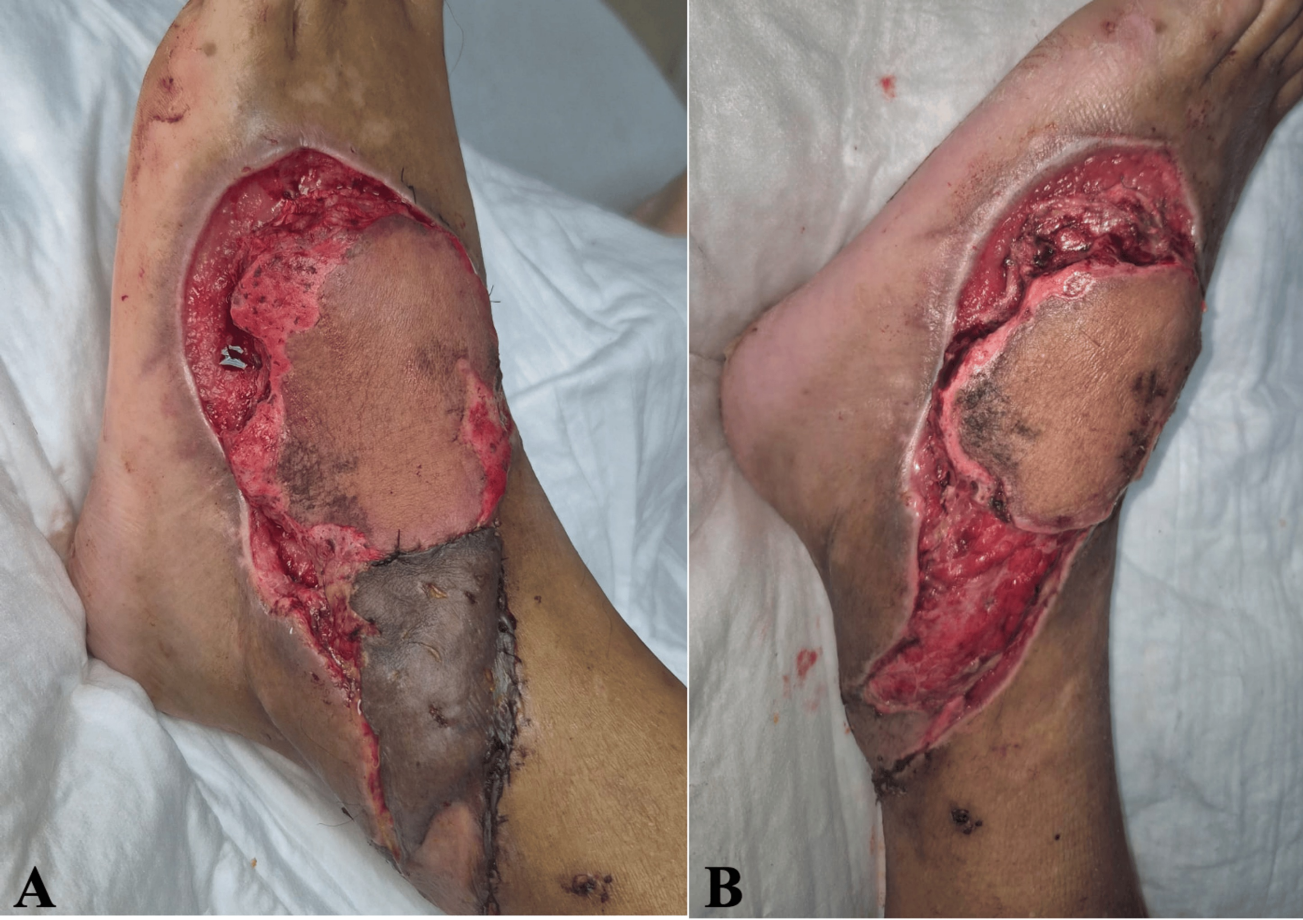
Progression of flap demarcation and debridement during the salvage protocol. (A) Postoperative day 10: enoxaparin administered every 24 hours. Tangential debridement of necrotic tissue was performed. (B) Postoperative day 17: debridement of residual devitalized tissue and continued advanced wound care were performed to achieve an optimal bed for definitive coverage.

Finally, between postoperative days 10 and 14, the enoxaparin dose was reduced to 10 mg every 24 hours, allowing the flap to develop adequate secondary neovascularization and consolidating the improvement in circulation (Figure 5A). In addition, tangential surgical debridement of necrotic tissue was performed while preserving viable underlying structures.

**Figure 6:**
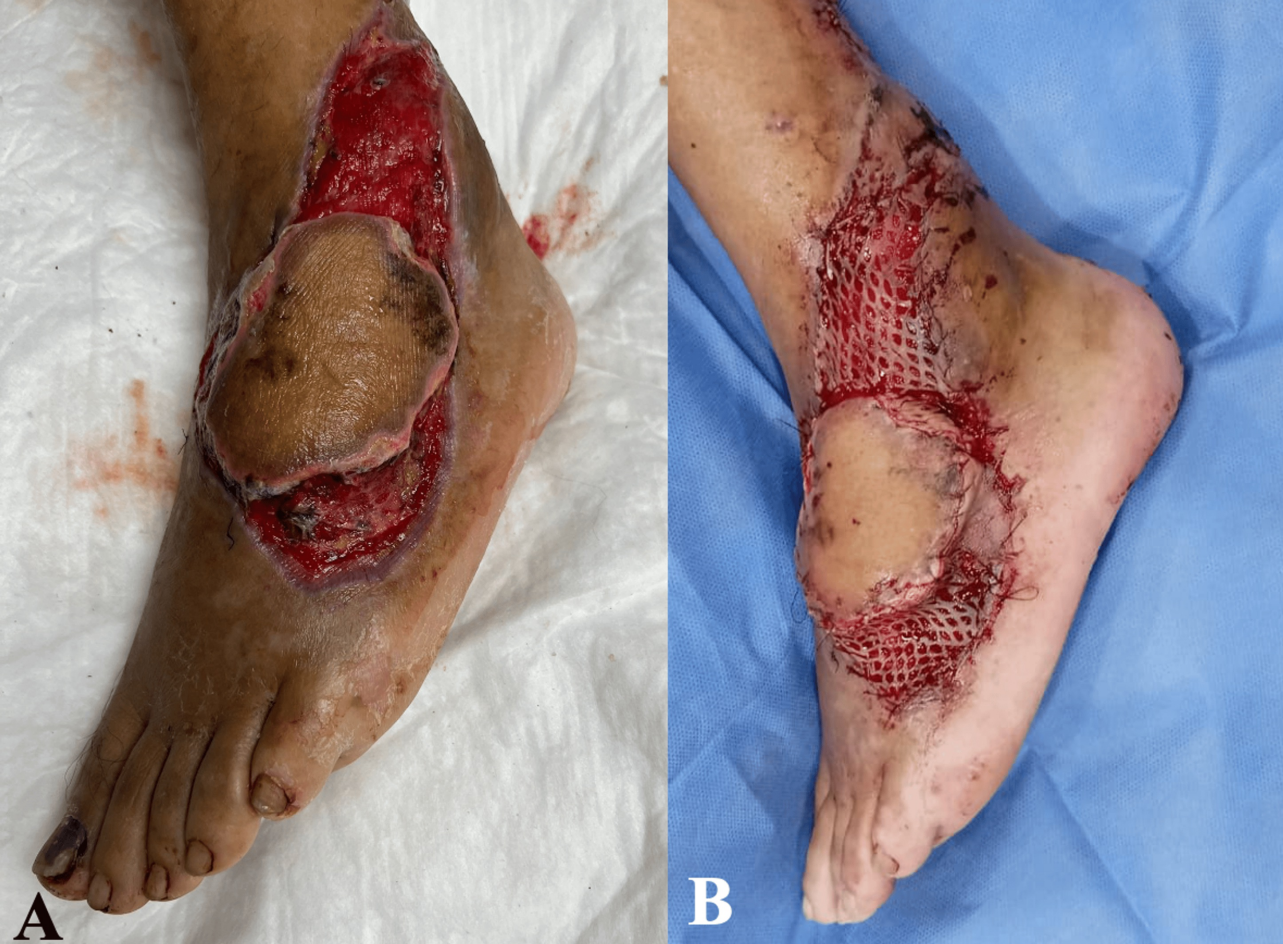
Preparation of the wound bed and definitive skin coverage. (A) Postoperative day 24: formation of healthy granulation tissue suitable for coverage of the residual raw area. (B) Postoperative day 28: definitive coverage with a split-thickness skin graft.

Throughout the treatment course, hemoglobin levels were closely monitored, and transfusions were administered when necessary to prevent flap hypoxemia. After the salvage period, the patient continued to receive periodic advanced wound care and mechanical debridement of devitalized tissue until an optimal wound bed for definitive coverage was achieved (Figures 5B, 6A).

**Figure 7:**
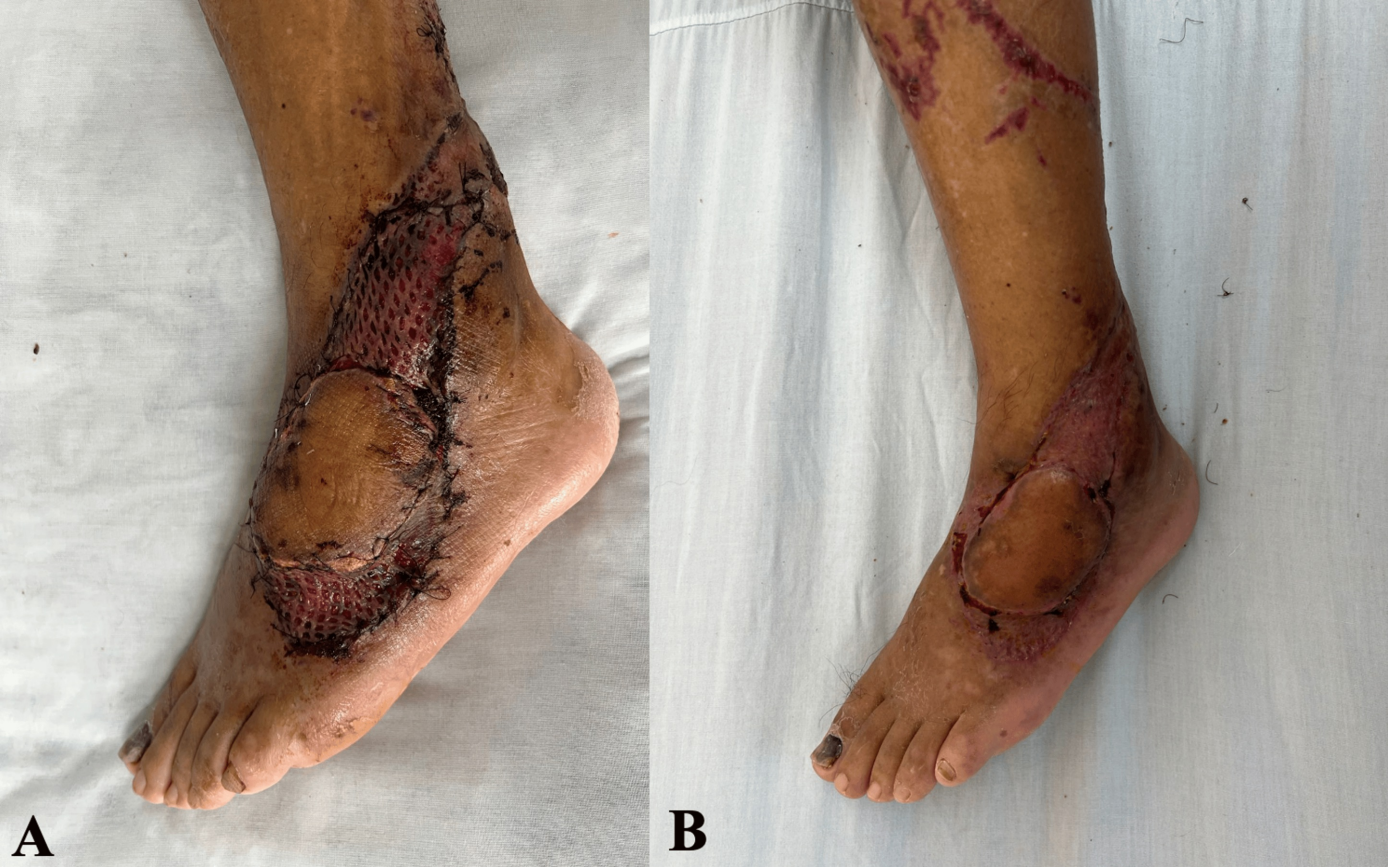
Follow-up of the skin graft after definitive coverage. (A) Postoperative day 32: graft evaluation showing 100% integration. (B) Postoperative day 53: complete graft integration with full epithelialization.

On postoperative day 28, surgical wound cleansing and definitive coverage with a meshed split-thickness skin graft were performed (Figure 6B). The graft was evaluated on postoperative day 32 after placement, showing 100% integration (Figure 7A). A final follow-up on postoperative day 53 demonstrated complete epithelialization (Figure 7B).

## Discussion

The reverse sural flap has variable success rates, generally ranging from 70% to 95%, depending on surgeon experience, the quality of the recipient site, and local conditions [1,4,5]. Reported complications include distal partial necrosis, wound dehiscence, retraction, and vascular compromise, with venous congestion being one of the most significant causes of flap loss [6,7].

Several factors may increase the risk of venous congestion in reverse sural flaps. Patient-related risk factors described in the literature include diabetes mellitus, peripheral vascular disease, smoking, and coagulation disorders [2,4]. In the present case, none of these systemic risk factors were identified.

However, flap-related and technical factors likely contributed to the development of venous congestion. The distally based design of the reverse sural flap relies on retrograde venous drainage through the superficial venous system, which is inherently susceptible to venous outflow impairment [5,6]. In addition, early postoperative edema and venous stasis in the distal lower extremity may further compromise venous return, particularly within the first 24 hours after surgery [6].

Pérez et al. reported a series of patients with venous congestion in whom a subset did not respond to surgical revision. These patients were subsequently treated with LMWH (enoxaparin), demonstrating that flaps managed under this protocol could be partially or completely salvaged [7].

Boissiere et al. conducted a systematic review of salvage techniques for venous congestion in flaps and identified multiple available strategies, including surgical re-exploration, venous drainage techniques, hirudotherapy, local heparin application, negative-pressure therapy, hyperbaric oxygen therapy, and others. Although the overall level of evidence remains low (case reports and small case series), the use of local anticoagulants appears to be a viable option when other methods fail or are not available [6].

Venous congestion is a common complication in distally based pedicled flaps, particularly in reverse sural flaps, which are frequently used for coverage of defects in the distal third of the leg and the foot [4,6]. Venous congestion compromises blood return and can rapidly lead to flap necrosis if not managed promptly within the first 24-48 postoperative hours [7]. In the present case, the use of local subcutaneous enoxaparin as a salvage strategy allowed adequate control of venous congestion and preservation of flap viability.

Venous congestion develops when venous outflow is disrupted, leading to the accumulation of blood within the flap, increased local venous pressure, and reduced clearance of carbon dioxide and metabolic byproducts. This creates a state of tissue hypoxia; hypoxia then promotes the release of inflammatory mediators and free radicals, which damage endothelial cells, impair microcirculation, and exacerbate vasodilation, ultimately perpetuating a vicious cycle of congestion. Additionally, venous stasis increases hydrostatic pressure and fluid transudation into the interstitium, resulting in edema that further worsens ischemia [6,7]. If not corrected early, the interruption of oxygen and nutrient supply leads to tissue necrosis. Common etiologies include pedicle torsion, insufficient venous drainage, venous thrombosis, and vascular spasm [4,5].

Management of venous congestion must be initiated promptly to prevent irreversible microcirculatory changes, tissue necrosis, and flap loss. When congestion is secondary to a mechanical cause, such as pedicle compression, immediate surgical exploration is essential to identify and correct the underlying issue, which may involve releasing compression or correcting pedicle torsion. However, when surgical revision is not possible or is unsuccessful, alternative therapeutic options exist. Medicinal leech therapy (hirudotherapy) [6,7] may promote venous drainage, but its availability is limited, and it carries risks such as infection, prompting exploration of additional salvage modalities. Local subcutaneous injection of LMWH has emerged as an effective option for managing venous congestion; this treatment reduces venous stasis and promotes neoangiogenesis, allowing the flap to recover its perfusion [7]. According to the protocol proposed by Pérez et al., treatment is initiated with high doses of local subcutaneous enoxaparin and progressively tapered depending on the flap's response until neovascularization is established [7].

In our case, application of this treatment resulted in a significant improvement in flap perfusion and a favorable clinical course, as demonstrated by the serial photographic documentation. On postoperative day 1, venous congestion was promptly identified; local subcutaneous enoxaparin administration progressively controlled the congestion, prevented extension of tissue necrosis, improved venous outflow, and facilitated neovascularization. These results are consistent with the findings reported by Pérez et al., who demonstrated that local subcutaneous enoxaparin can partially or completely salvage flaps with venous congestion, and with the systematic review by Boissiere et al., who identified local heparin injection as one of the most effective non-surgical salvage methods available [6,7].

Despite these positive outcomes, several areas require further investigation, including the standardization and evaluation of optimal dosing, treatment duration, and the identification of potential long-term adverse effects. As a single case report, the findings cannot be generalized, and causal conclusions regarding the superiority of this approach over other salvage methods cannot be established. Additionally, the absence of objective perfusion measurements and the lack of long-term comparative outcomes limit the ability to fully assess efficacy. Larger case series and comparative studies are required to further validate this protocol. Additionally, more comparative studies are needed to assess the effectiveness of enoxaparin relative to other salvage methods, such as hirudotherapy or negative-pressure therapy, to determine the most appropriate intervention based on patient-specific conditions and flap characteristics.

## Conclusions

The use of local subcutaneous enoxaparin is an effective and accessible strategy for managing venous congestion in pedicled flaps. Early and systematic implementation of this approach, combined with timely and adequate debridement of necrotic tissue and continuous postoperative monitoring, has proven to be essential for ensuring flap survival. This therapeutic method is particularly valuable in settings with technical and economic limitations, where traditional surgical options may not be feasible. The existing literature supports its effectiveness, and the case presented here further demonstrates and reinforces its applicability for future reconstructive surgical interventions. 
